# Cellulosome Localization Patterns Vary across Life Stages of Anaerobic Fungi

**DOI:** 10.1128/mBio.00832-21

**Published:** 2021-06-01

**Authors:** Stephen P. Lillington, William Chrisler, Charles H. Haitjema, Sean P. Gilmore, Chuck R. Smallwood, Vaithiyalingam Shutthanandan, James E. Evans, Michelle A. O’Malley

**Affiliations:** a Department of Chemical Engineering, University of California, Santa Barbara, Santa Barbara, California, USA; b Environmental Molecular Sciences Laboratory, Pacific Northwest National Laboratory, Richland, Washington, USA; c School of Biological Sciences, Washington State University, Pullman, Washington, USA; d Bioscience Division, Department of Molecular and Microbiology, Sandia National Laboratories, Albuquerque, New Mexico, USA; University of California, Berkeley

**Keywords:** anaerobic fungi, antibody, cellulase, cellulosome, enzyme, immunofluorescence

## Abstract

Anaerobic fungi (*Neocallimastigomycota*) isolated from the guts of herbivores are powerful biomass-degrading organisms that enhance their degradative ability through the formation of cellulosomes, multienzyme complexes that synergistically colocalize enzymes to extract sugars from recalcitrant plant matter. However, a functional understanding of how fungal cellulosomes are deployed *in vivo* to orchestrate plant matter degradation is lacking, as is knowledge of how cellulosome production and function vary throughout the morphologically diverse life cycle of anaerobic fungi. In this work, we generated antibodies against three major fungal cellulosome protein domains, a dockerin, scaffoldin, and glycoside hydrolase (GH) 48 protein, and used them in conjunction with helium ion and immunofluorescence microscopy to characterize cellulosome localization patterns throughout the life cycle of Piromyces finnis when grown on simple sugars and complex cellulosic carbon sources. Our analyses reveal that fungal cellulosomes are cell-localized entities specifically targeted to the rhizoids of mature fungal cells and bodies of zoospores. Examination of cellulosome localization patterns across life stages also revealed that cellulosome production is independent of growth substrate in zoospores but repressed by simple sugars in mature cells. This suggests that further exploration of gene regulation patterns in zoospores is needed and can inform potential strategies for derepressing cellulosome expression and boosting hydrolytic enzyme yields from fungal cultures. Collectively, these findings underscore how life cycle-dependent cell morphology and regulation of cellulosome production impact biomass degradation by anaerobic fungi, insights that will benefit ongoing efforts to develop these organisms and their cellulosomes into platforms for converting waste biomass into valuable bioproducts.

## INTRODUCTION

Anaerobic fungi (phylum *Neocallimastigomycota*) are commonly found in the digestive tracts of large, herbivorous animals, where they play an important role in colonizing and degrading ingested plant biomass (1, 2). Despite accounting for only ∼8% by mass of the gut microflora (3), reducing the ruminal anaerobic fungi population has been shown to cause a 49 to 70% decrease in unpretreated biomass consumption by sheep and cattle compared to those with a natural abundance of fungi in the rumen (4, 5). Furthermore, anaerobic fungi have been shown to preferentially degrade the more recalcitrant, lignin-rich plant matter that cellulolytic rumen bacteria cannot catabolize, making them attractive potential hosts for converting unpretreated waste biomass into high-value bioproducts (1, 6). Since the first description of these organisms in 1975 by Colin Orpin (7), more than 28 species of anaerobic fungi have been isolated and characterized to better understand both their ecological role in the rumen microbiome and their biomass-degrading machinery (8, 9).

Genomic and transcriptomic sequencing have shown that anaerobic fungi harbor a wealth of carbohydrate-active enzymes (CAZymes) that enable their deconstruction of crude lignocellulose. Whole-genome sequencing of 6 species of anaerobic fungi (10–13) revealed that they encode on average over 4-fold more CAZymes than Trichoderma reesei and Aspergillus niger, the sources of the most popular cellulolytic cocktails in industry (https://mycocosm.jgi.doe.gov) (14). The functional diversity of the encoded gut fungal CAZymes is similarly astounding; represented among the published genomes of *Neocallimastigomycota* are 43 glycoside hydrolase (GH) families, 28 glycosyl transferase (GT) families, 9 carbohydrate esterase (CE) families, 5 polysaccharide lyase (PL) families, and 19 carbohydrate binding module (CBM) families (https://mycocosm.jgi.doe.gov). While their genomic potential is impressive, more biochemical data and functional knowledge of how this diverse group of hydrolytic enzymes function *in vivo* are necessary before the degradative machinery of anaerobic fungi or the organisms themselves can be developed into useful platforms for the conversion of waste biomass.

Like several species of anaerobic bacteria, anaerobic fungi incorporate many of these CAZymes into multienzyme complexes called cellulosomes, which colocalize lignocellulolytic enzymes of complementary function to greatly enhance degradative activity (15). Fungal cellulosomes are thought to mimic bacterial ones in their general structure, in which modular dockerin domains attached to catalytic proteins noncovalently bind repeated cohesin domains on a central, membrane-anchored scaffoldin. These interacting parts have been well characterized in bacterial cellulosomes; the molecular details of their interaction are well described, as is their localization on the cell surface *in vivo* (16, 17). As a result of its characterization, the bacterial cellulosome has served as a template for designing synthetic protein complexes with wide-ranging applications in nanobiotechnology, recently reviewed in reference 15. In contrast, while the fungal dockerin domain has a known sequence and structure divergent from the bacterial dockerin (18), the identity of a conserved cohesin domain in anaerobic fungi remains elusive, though a conserved group of large (>500 kDa), repeat-rich scaffoldins with no sequence homology to any bacterial cellulosome component was recently shown to bind recombinant fungal dockerin with a *K_D_* (equilibrium dissociation constant) of 994 nM (10). No *in vivo* colocalization studies have cemented these scaffoldins’ role in native fungal cellulosomes yet, but comparative genomic analyses of fungal cellulosome composition and domain architecture suggest that fungal cellulosome parts may serve as better templates than bacterial ones for certain synthetic systems. An observed roadblock to constructing designer cellulosomes using bacterial components is failure to construct stable, functional enzyme-dockerin chimeras, particularly for enzymes not of bacterial origin or for N-terminal dockerin fusions (19). In contrast to bacterial dockerins, fungal dockerin domains are observed as N- and C-terminal fusions to enzymes, and the diversity of dockerin-containing proteins in anaerobic fungi significantly exceeds that of bacteria (10), suggesting more general compatibility of fungal cellulosome components in the construction of synthetic complexes. Where cellulosomes localize in native systems and how these structures attach to cells and biomass are key unresolved details to inform the successful design of synthetic fungal cellulosomes. No previous work has investigated the *in vivo* localization of the newly discovered fungal scaffoldins, and only one study observed dockerin localization on the cell surface of *Orpinomyces* sp. strain PC-2 using electron microscopy and an immunogold-labeled anti-dockerin antibody (20).

**FIG 1:**
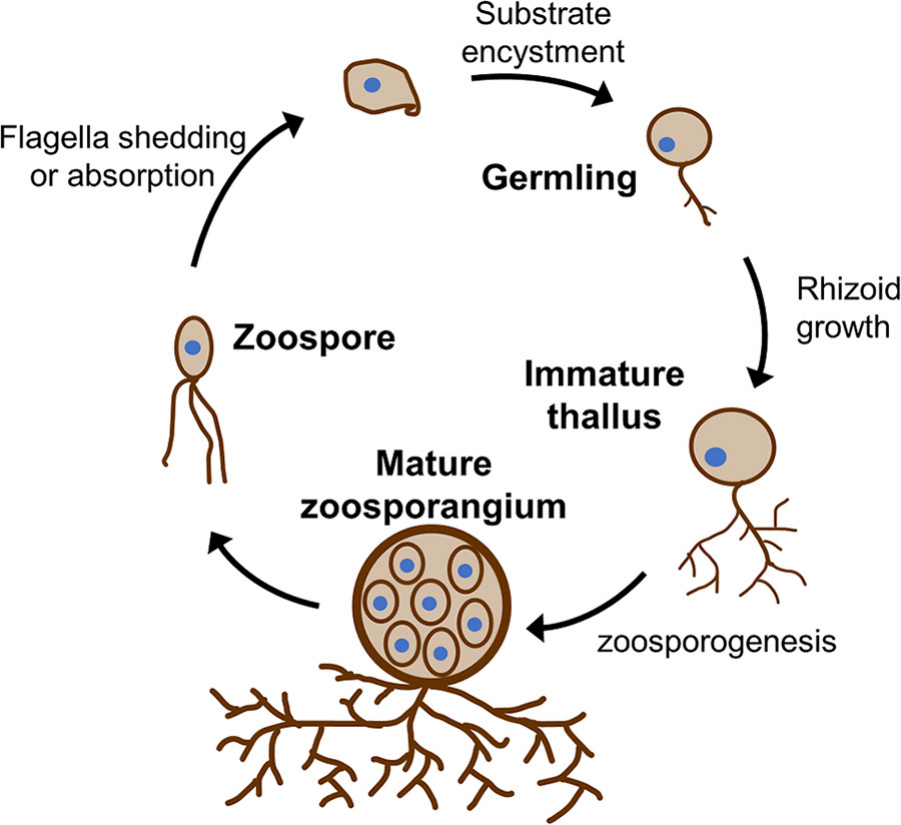
Anaerobic fungi replicate through a multistep life cycle accompanied by dramatic morphological change. Motile, flagellated zoospores follow chemiosmotic gradients to a carbon source and, on insoluble substrates, encyst themselves through the extension of root-like rhizoids into the substrate. The main zoospore body simultaneously matures into a thallus that, in the reproductive stage of growth, hosts newly formed zoospores that eventually break out of the zoosporangium to repeat the growth cycle. This schematic represents reproduction of a monocentric anaerobic fungus with multiflagellar zoospores.

Deriving insight into the cellulosome’s functional role by observing its spatial localization patterns is complicated by the complex life cycle of anaerobic gut fungi, since the composition and role of cellulosome-associated proteins (10) likely change during fungal life cycle progression. As close relatives to *Chytridiomycota*, anaerobic fungi proliferate through a life cycle in which a motile zoospore encysts on plant biomass, growing root-like rhizoids and maturing into a zoospore-filled sporangium that releases more zoospores (Fig. 1). The drastic morphological changes seen during life cycle progression raise the question of which life stage is predominantly responsible for rapid lignocellulose hydrolysis. Furthermore, as these organisms are potential ingredients in lignocellulolytic enzyme cocktails, it is important to understand how production of cellulosomes is regulated during life cycle progression. It has generally been assumed that the rhizoid-bearing, maturing cells are responsible for both robust cellulosome production and rapid lignocellulose hydrolysis, since rhizoids bear a resemblance to hyphae, the primary sites of enzyme secretion and biomass hydrolysis in filamentous fungi like Aspergillus niger (21). Experimental evidence confirming the functional importance of mature cells or their rhizoids to biomass hydrolysis is still lacking.

In this work, we developed imaging probes unique to fungal cellulosomes that label key cellulosome protein domains in the anaerobic fungus Piromyces finnis. Antibodies raised against the fungal scaffoldin, dockerin, and GH48 domain, the most abundant dockerin-fused enzyme in the *P. finnis* cellulosome, enabled localization of these domains in samples from two different genera within *Neocallimastigomycota.* Using these tools, we show that rhizoids are the dominant location of lignocellulose hydrolysis in mature cells, displaying high coverage of both GH48 and dockerin domains localized to the surface, but only when anaerobic fungi are challenged with insoluble, complex carbon sources such as Whatman cellulose paper. In contrast, zoospores display both GH48 and dockerin-containing proteins on their main body regardless of growth substrate complexity, insinuating differential regulation of cellulosomal CAZyme production throughout the fungal life cycle. Our results suggest that zoospores accompany rhizoid-bearing cells as important biomass degraders in the rumen environment. They also imply that comparative analysis of zoospore and mature cell gene expression data may yield genetic targets to engineer anaerobic fungi for overproduction of CAZymes and cellulosomes throughout the fungal life cycle under all growth conditions (22). These first insights into cellulosome spatial localization and life cycle-dependent regulation of cellulosome production will benefit both future laboratory study seeking to engineer fungal cellulosomes and future development of anaerobic fungi into platforms for waste biomass upcycling or hydrolytic enzyme production.

## RESULTS

### Anaerobic fungi use rhizoids to penetrate and disrupt plant biomass.

A key characteristic of rhizoid-forming anaerobic fungi is the highly branched morphology of mature fungal cells, which is unique among members of the rumen microbiota. In the rumen, root-like rhizoids are known to facilitate biomass colonization by anaerobic fungi and are hypothesized to play a major role in lignocellulose hydrolysis, both by mechanically disrupting ingested biomass (6, 23, 24) and by attaching fungal cellulosomes, the primary source of cellulolytic power (25–28). To our knowledge, neither of these assumptions has been explicitly verified for any anaerobic fungal isolate. Prior works report size estimates for fungal cellulosomes based on size exclusion chromatography and electron microscopy ranging from 700 kDa to tens of megadaltons (20, 25, 26), suggesting that these structures might be visualized by helium ion microscopy (HIM). We sought to determine whether fungal rhizoids host fungal cellulosomes using HIM analysis of anaerobic fungi growing on lignocellulosic substrates.

**FIG 2:**
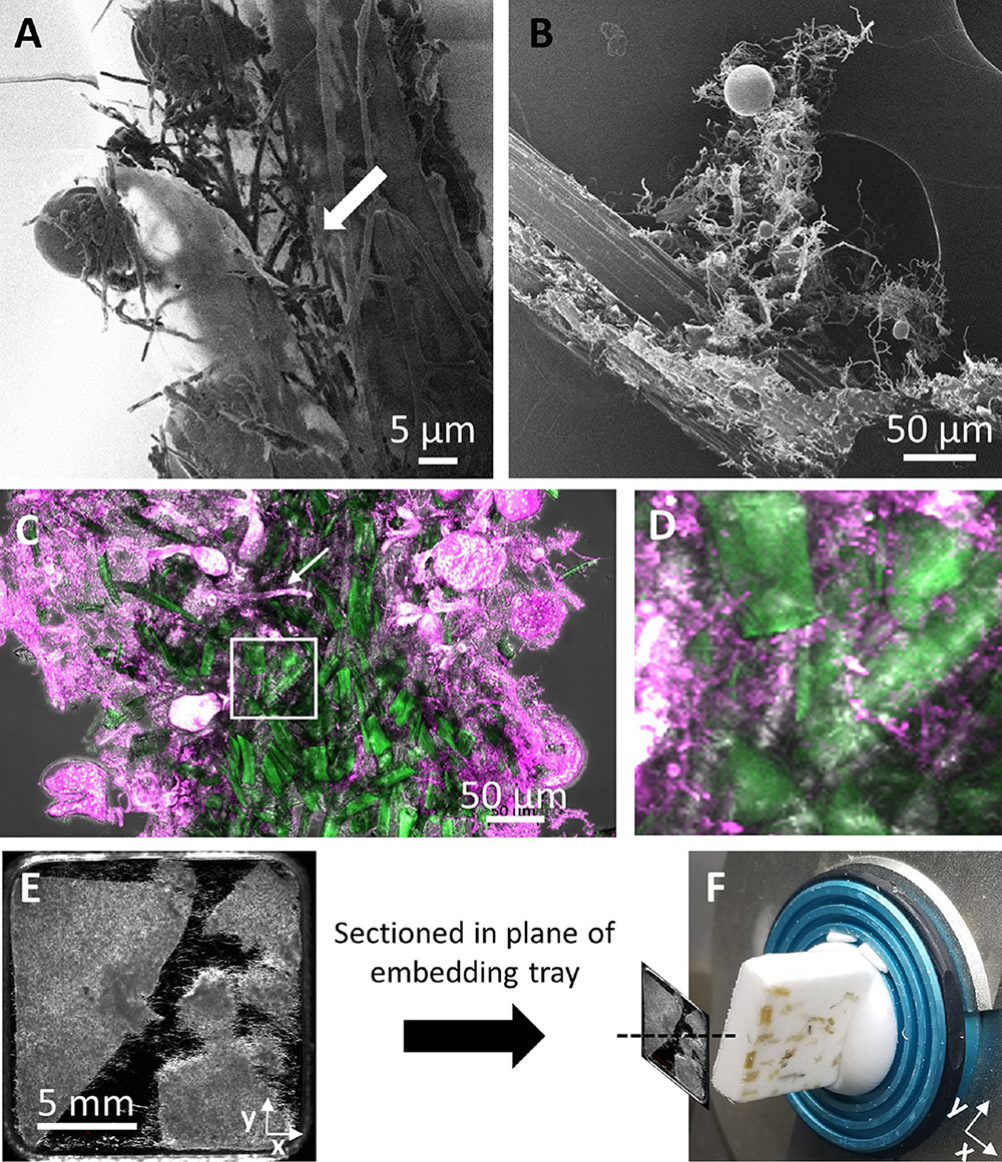
Rhizoids mediate substrate attachment and penetration by mature *Piromyces* cells. Helium ion micrographs show that rhizoids of mature *Piromyces finnis* cells mediate contact with the lignocellulosic substrate and suggest a penetrative growth phenotype, indicated by the arrow, that aids in fungal biomass degradation (A and B). Substrate penetration by rhizoids was confirmed by cryosectioning a formaldehyde-fixed culture of *Piromyces finnis* grown on cellulose paper, showing clear growth into the substrate interior, including one rhizoid, identified by the arrow, penetrating over 100 μm (C). A magnified portion of the maximum-intensity projection (white box in panel C) highlights the extent to which the rhizoid network colonizes the substrate interior (D). Sections were prepared by cutting in the plane of the embedded sample (E and F). Confocal micrographs from 33 2-μm z-steps were obtained with an LD C-Apochromat 40×/1.1 W Korr M27 objective and collapsed to a maximum-intensity projection using Zen software (Zeiss). FM 1-43 membrane stain (pink) and SYBR gold (green) were used.

Lower-magnification HIM micrographs of *Piromyces finnis* grown on dried switchgrass biomass demonstrate that the rhizoids of mature cells are the major interface with grass particles (Fig. 2A and B). Rhizoidal morphology greatly increases the interfacial surface area between cells and their carbon source, likely an important factor in enhancing lignocellulose hydrolysis. Though HIM is a surface microscopy technique and cannot resolve internal structures, the apparent growth of rhizoids into the grass particles in Fig. 2A and B suggests that these structures penetrate the colonized substrate to access trapped carbon. This phenomenon is well documented in samples taken from a live animal rumen, showing heavy internal rhizoid colonization of damaged, living plant tissues (6, 29), but is not confirmed to occur during anaerobic fungal growth on dried (ligno)cellulosic substrates, which are the recommended biomass source for biorefinery concepts (30). To determine if biomass colonization patterns are similar on dried substrates, we cryosectioned and imaged by confocal microscopy formaldehyde-fixed samples of an anaerobic fungus growing on Whatman cellulose paper (Fig. 2E and F). Though sacrificing in resolution using confocal instead of helium ion microscopy, the sectioned samples enabled unequivocable observation of rhizoid penetration into the substrate interior, with one rhizoid shown growing over 100 μm into the substrate (Fig. 2C and D).

Without coincident immunolabeling, it was difficult to identify cellulosome structures in the HIM micrographs directly, though we frequently observed bumpy regions in what appeared to be highly branched rhizoid systems with globular features 10 to 100 nm in diameter (see Fig. S1 in the supplemental material). It is important to note that biomass-only negative controls possessed somewhat similar fibrillar networks of a similar length scale and a generally wispy microstructure that obfuscated which structural features arose from cells versus the substrate (Fig. S3). As such, it is possible that Fig. S1 depicts enzyme-degraded reed canary grass, with the highly branched fibrillar network comprising reed canary grass filaments and the globular structures possibly representing bound proteins or cellulosomes. However, HIM micrographs in which rhizoids were clearly emerging from sporangia definitively highlighted rhizoids’ “rough” surfaces, possibly characteristic of surface-displayed cellulolytic enzymes and/or cellulosomes that may localize to these structures (Fig. S2). Such structural features could have been artifacts of chemical fixation during sample preparation, and as such, these data were used solely to generate hypotheses about the localization of fungal cellulosomes *in vivo*.

10.1128/mBio.00832-21.2 FIG S1 Potential cellulosome structures from *Piromyces finnis* visualized by helium ion microscopy (HIM). Images were obtained from fixed, dehydrated samples of *P. finnis* grown on reed canary grass as described in Materials and Methods. Globular structures with diameters in the 10-nm to 100-nm range, consistent with megadalton-sized protein complexes, are apparent on the surfaces of filamentous structures ∼10 nm in diameter. Filaments this small could possibly originate from the reed canary grass substrate, in which case the globular structures may be bound, cell-free cellulosome complexes. Download FIG S1, DOCX file, 0.6 MB. Copyright © 2021 Lillington et al. 2021 Lillington et al. https://creativecommons.org/licenses/by/4.0/ This content is distributed under the terms of the Creative Commons Attribution 4.0 International license.

10.1128/mBio.00832-21.3 FIG S2 Larger rhizoids of *Piromyces finnis* and *Neocallimastix californiae* cells have rough surfaces covered with globular structures that may be proteins or protein complexes. (A to C) Micrographs from *P. finnis* cultured on reed canary grass. (B) Magnification of part of the rhizoid annotated in panel A. Download FIG S2, DOCX file, 1.8 MB. Copyright © 2021 Lillington et al. 2021 Lillington et al. https://creativecommons.org/licenses/by/4.0/ This content is distributed under the terms of the Creative Commons Attribution 4.0 International license.

10.1128/mBio.00832-21.4 FIG S3 Reed canary grass from culture tubes not inoculated with anaerobic fungi show a wispy, complex microstructure by helium ion microscopy. (A) Fibrillar microstructures with diameters around 10 nm are present in biomass-only control samples, making it more difficult to identify potential parts of fungal cells or cellulosomes at this length scale. (B to D) Larger, cylindrical structures that resemble anaerobic fungal rhizoids also appear in the biomass-only control, emphasizing both the potential uncertainty in identifying subcellular structures from HIM images and the need to identify rhizoids as structures emerging from sporangia, which are clearly observed only in the fungus-containing samples. Download FIG S3, DOCX file, 2.1 MB. Copyright © 2021 Lillington et al. 2021 Lillington et al. https://creativecommons.org/licenses/by/4.0/ This content is distributed under the terms of the Creative Commons Attribution 4.0 International license.

### Rhizoids of mature fungal cells display cellulosomes and other lignocellulolytic proteins.

**FIG 3:**
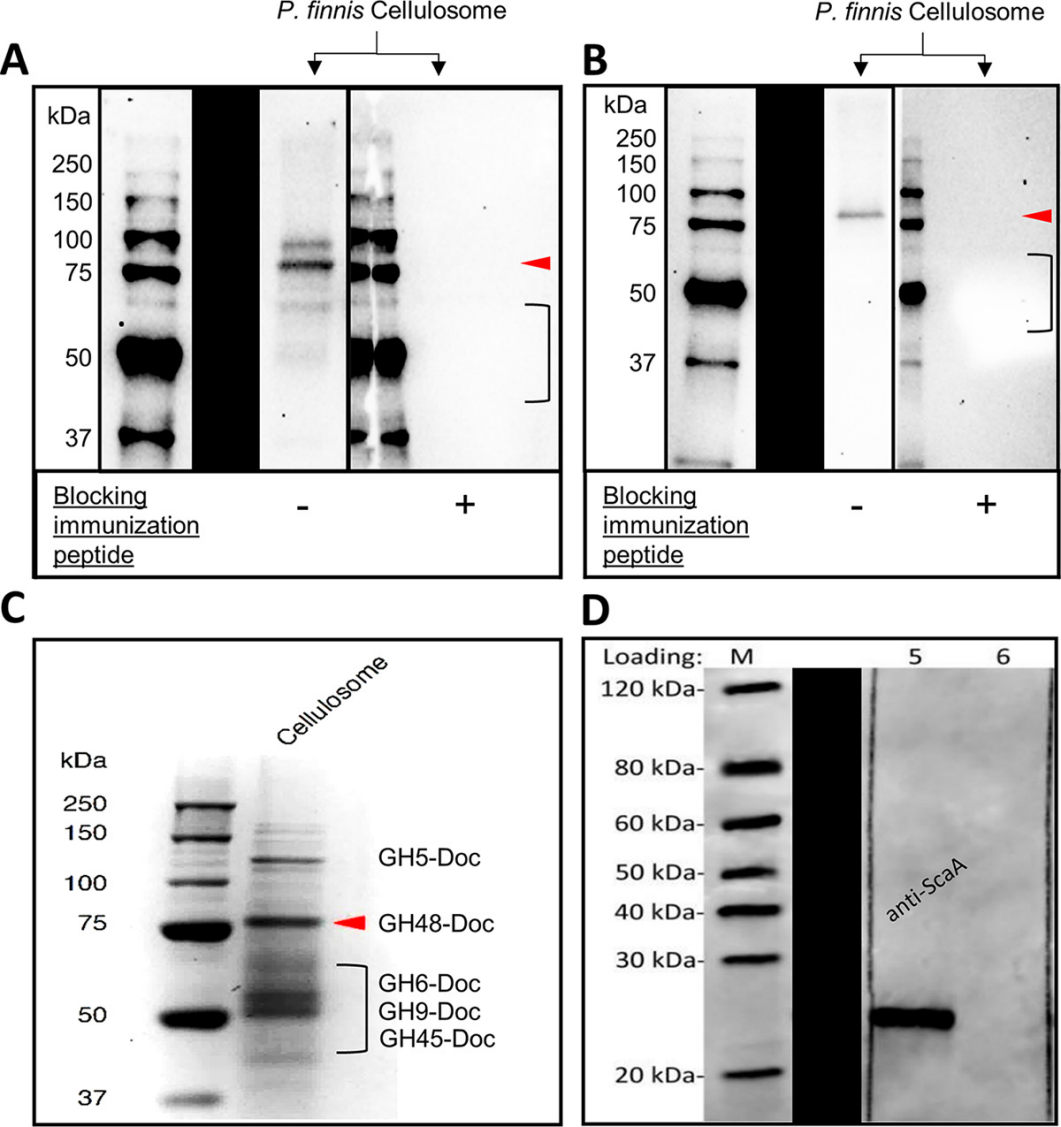
Antibodies generated against recombinant cellulosome protein fragments bind their expected targets in the native *P. finnis* cellulosome. (A) Antibody raised against a synthetic peptide fragment from a *P. finnis* dockerin domain binds to multiple proteins from the *P. finnis* cellulosome. Preincubating the antibody with the immunizing peptide ablates labeling of any cellulosome protein, demonstrating high antibody-epitope specificity. (B) Antibody raised against a synthetic peptide fragment from a *P. finnis* GH48 catalytic domain appears to bind a single target in *P. finnis* cellulosome. Preincubating the antibody with the immunizing peptide ablates labeling of any cellulosome protein, demonstrating high antibody-epitope specificity. (C) Corresponding Coomassie blue-stained SDS-PAGE gel of the *P. finnis* cellulosome. Putative band identities annotated by arrows or brackets in panels A and B were assigned from qualitative MS proteomics data provided in reference 10. “Doc” indicates that the protein contains at least one fused dockerin domain. The annotations provide evidence that the antibodies label the expected proteins, as many dockerin-containing protein bands are present in panel A, and only the GH48-Doc band is present in panel B. (D) A Western blot of mouse monoclonal antibody against a recombinant fragment of the *P. finnis* ScaA scaffoldin protein shows specific binding to the scaffoldin fragment. As in panels A and B, a black bar separates nonadjacent lanes from the same SDS-PAGE gel for clarity. No binding to any protein in the *P. finnis* cellulosome in panel C was observed.

To determine whether the putative rhizoid-localized proteins were cellulosomes, antibodies were raised against three recombinantly produced proteins from *Piromyces finnis*: a double dockerin domain, which is the prevailing form in cellulosome-associated proteins (10), a fragment of the dockerin’s putative binding partner ScaA, and a CAZyme of the GH48 family, the dominant enzyme in the *Piromyces* cellulosome (31, 32). Western blots show that the anti-dockerin and anti-GH48 antibodies bind to multiple proteins from the *Piromyces finnis* cellulosome, several of which were verified to contain the antibodies’ target domain by mass spectrometry (MS) proteomics (10) (Fig. 3A and B). The anti-ScaA antibody bound specifically to the recombinant fragment used for immunization (Fig. 3C) but did not appear to bind any protein from the *P. finnis* cellulosome in a Western blot. One possible explanation is that, though MS proteomics confirmed the presence of ScaA in the *P. finnis* cellulosome (10), the antigen’s abundance may have been below the detection limit of standard Western blotting (data not shown). As a secreted protein, native ScaA is also expected to be posttranslationally modified in ways the recombinant protein was not, which may provide an alternative explanation for this observation if the anti-ScaA epitope is posttranslationally modified. An additional possibility is that the antibody binding site may not be accessible in an *in vivo* context, being either buried in the core of the full-length ScaA or blocked by the binding of other peptides.

**FIG 4:**
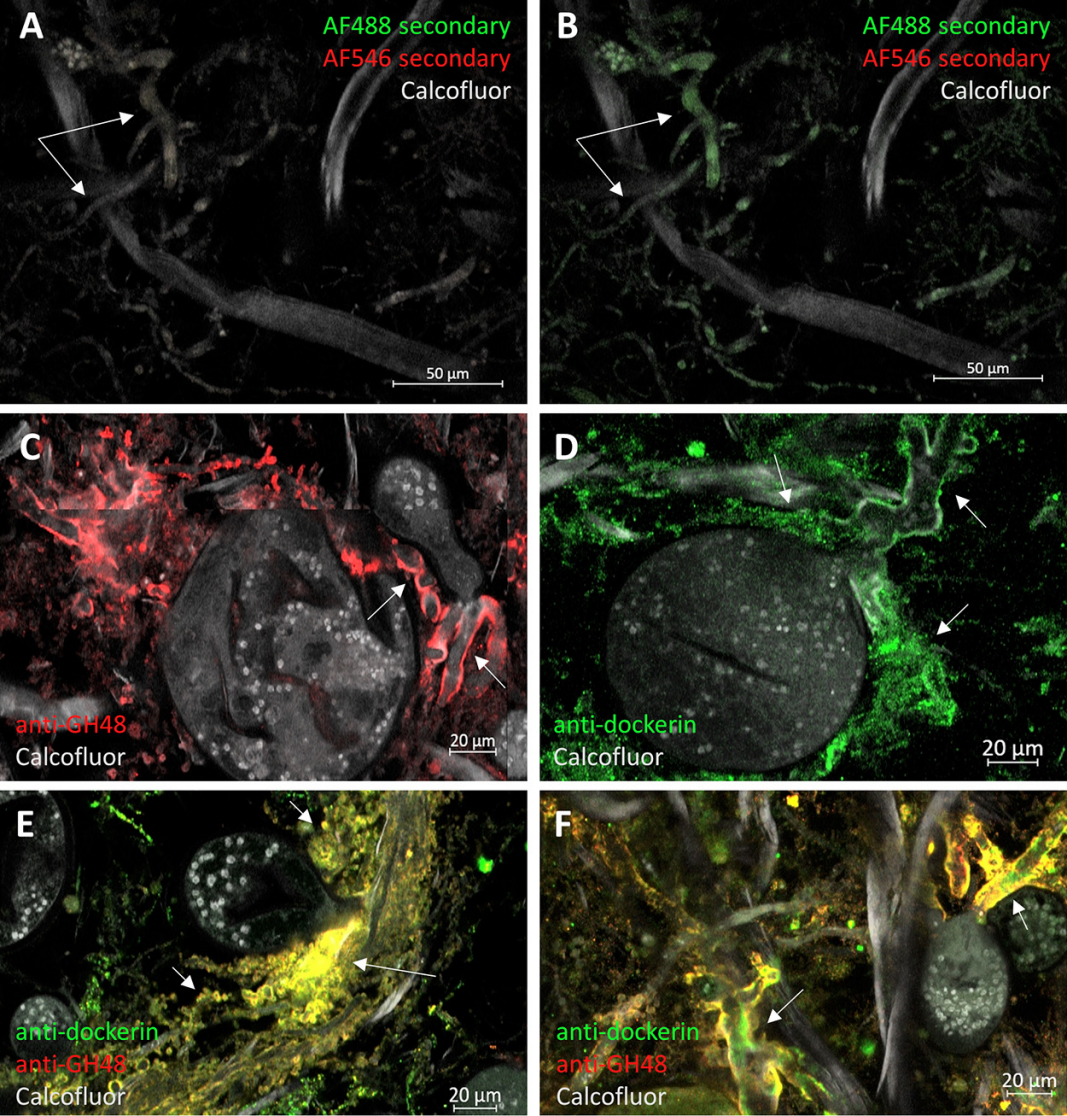
Mature *Piromyces finnis* cells localize cellulose-degrading machinery to rhizoids when growing on complex substrates. Immunofluorescence staining of fixed *P. finnis* cells grown on Whatman filter paper shows specific, intense localization of anti-GH48 (red) and antidockerin (green) signal to rhizoids (arrows) (C to F). A negative control stained only with calcofluor white and secondary antibodies (donkey anti-rabbit IgG–Alexa Fluor 488, donkey anti-rabbit IgG–Alexa Fluor 546, and goat anti-mouse IgG–Alexa Fluor 647) shows low background signal as expected (A and B). Antidockerin labeling was consistently lower than anti-GH48 labeling across samples, and the different image display settings used to produce panels C and D are reproduced in panels A and B, respectively. (E and F) Maximum-intensity projections from five 1-μm confocal image stacks that illustrate the abundant colocalization of anti-dockerin and anti-GH48 signal to cell rhizoids, as evidenced by apparent yellow staining caused by coemission of red and green light. Each panel except panels A and B, which are the same micrograph with different image processing, is representative of three technical replicate samples from a single culture tube. The negative controls are representative of five technical replicates. The same display settings and gamma parameters in Zen (Zeiss Microscopy) were used to generate panels A, C, E, and F. Different but consistent display settings and gamma parameters were used to generate panels B and D. Confocal micrographs were obtained with an LD C-Apochromat 40×/1.1 W Korr M27 objective. Antibodies and stains used: calcofluor white (gray); for GH48, rabbit anti-GH48 (primary) and donkey anti-rabbit IgG–Alexa Fluor 546 (secondary; red); for dockerin, rabbit antidockerin (primary) and donkey anti-rabbit IgG–Alexa Fluor 488 (secondary; green).

We characterized dockerin and GH48 localization patterns by analyzing formaldehyde-fixed cultures of *Piromyces finnis* after 72 to 96 h of growth on Whatman cellulose paper. Whatman paper was chosen as a substrate both because it is known to induce robust production of CAZymes and dockerin-containing proteins in anaerobic fungi (32) and because, in addition to fungal cell walls, it is also stained by calcofluor white, facilitating visualization. Samples were stained with calcofluor white and one or more of the three antibodies before visualization under a Zeiss LSM 710 confocal microscope equipped with an LD C-Apochromat 40×/1.1 W objective. As shown in Fig. 4, both anti-dockerin and anti-GH48 signals localize intensely to the surface of mature fungal cell rhizoids during growth on cellulose paper, consistent with the HIM images depicting protein-like structures on rhizoid surfaces (Fig. S1 and S2). Surface-localized dockerin and GH48 appear on all parts of a cell’s rhizoid system, both at the oldest growth near the thallus or sporangial head and at the newer, more highly branched growth (Fig. 4C to F). The absence of signal in samples stained only with secondary antibodies provides strong evidence these observations are not an artifact of our visualization method (Fig. 4A and B). Importantly, no anti-dockerin or anti-GH48 signal localized to thallus or sporangial heads, suggesting that rhizoids are the primary site for lignocellulose hydrolysis. The anti-ScaA antibody did not yield specific positive staining comparable in appearance to that of the dockerin or GH48 in any part of the cells. Much of the anti-ScaA signal appeared nonspecifically bound to cellulose paper fibrils, suggesting that the anti-ScaA antibody likely does not bind its intended target.

### Growth conditions control production of key cellulosome proteins across the life stages of anaerobic fungi.

**FIG 5:**
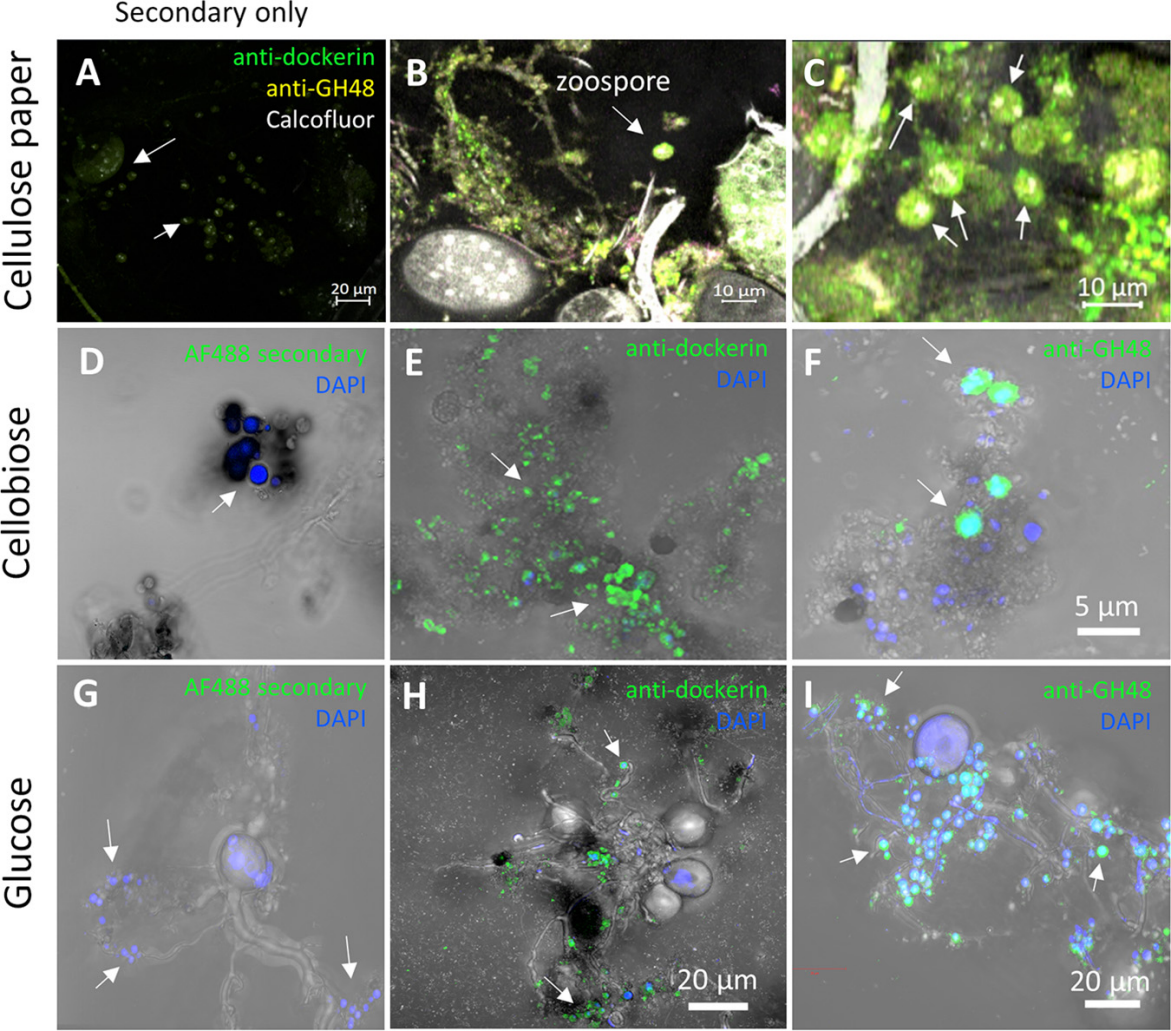
Zoospores actively express and display cellulosome proteins during growth on simple and complex substrates. Micrographs of zoospores from samples of *P. finnis* grown on Whatman paper show intense antidockerin and anti-GH48 signal (B and C) relative to a secondary-antibody-only control (A). Representative zoospores are labeled with arrows in all images. Panels A through C were all processed by Zen software using the same display settings and gamma parameters. During growth on cellobiose, antidockerin and anti-GH48 signals appear uniquely restricted to *P. finnis* zoospores with no staining of mature cells (E and F). *Neocallimastix californiae*, another monocentric anaerobic fungus, demonstrates the same staining pattern when grown on glucose, suggesting that substrate-dependent cellulosome rhizoid display and substrate-independent zoospore cellulosome display are general phenotypes of monocentric anaerobic fungi (H and I). (D and G) Negative controls stained only with donkey anti-rabbit IgG–Alexa Fluor 488. The images are representative of at least two technical replicates per carbon source, with one biological replicate for cellulose paper cultures and two biological replicates for cellobiose and glucose cultures. All confocal micrographs were obtained with an LD C-Apochromat 40×/1.1 W Korr M27 objective. Antibodies and stains used: Calcofluor white (gray) (A to C) and DAPI (4′,6-diamidino-2-phenylindole; blue) (D to I); for GH48, rabbit anti-GH48 (primary) and anti-rabbit IgG–Alexa Fluor 546 (secondary; yellow) (A to C) or anti-rabbit IgG–Alexa Fluor 488 (secondary; green) (F and I); for dockerin, rabbit anti-dockerin (primary) and anti-rabbit IgG–Alexa Fluor 488 (secondary; green).

The HIM and initial immunofluorescence micrographs suggested that fungal rhizoids primarily serve as the sites for cellulosome localization. However, further examination of immunofluorescence images from the *Piromyces* filter paper cultures showed an abundance of small spherical bodies consistent in size with zoospores (∼5 μm in diameter) that also displayed high dockerin and GH48 detection signal well beyond that seen in the negative control (Fig. 5A to C).

The apparent presence of surface-displayed cellulosomes on both zoospores and mature fungal thalli is surprising given the stark difference in cell morphology and previously described roles of the two life stages, in which rhizoid-bearing thalli are the chief biomass degraders, while motile zoospores search for new carbon sources to colonize (33). These results also imply an interesting connection between cellulosome localization and progression through the fungal life cycle, in which localization shifts from the zoospore body during the substrate search to the growing rhizoids after substrate encystment.

Since cellulosome and CAZyme expression is highly dependent on the growth substrate (32), we hypothesized that dockerin and GH48 localization patterns would change as a function of substrate complexity. To test this hypothesis, we stained formaldehyde-fixed samples of *Piromyces finnis* grown on the soluble disaccharide cellobiose for 72 to 96 h with anti-dockerin and anti-GH48 antibodies. Intriguingly, zoospores remained hot spots for dockerin and GH48 signal under these conditions, but the rhizoids of thalli appeared devoid of both proteins (Fig. 5E and F). The same patterns were observed for glucose-grown samples of a different anaerobic fungus, Neocallimastix californiae (Fig. 5H and I), indicating that the unique expression and display of cellulosomal CAZymes by zoospores during growth on soluble sugars may be general to monocentric anaerobic fungi. Consistent with the immunofluorescence observations, HIM micrographs of *N. californiae* zoospores from cultures grown on both glucose and corn stover also indicated the presence of surface-displayed proteins, as evidenced by the clustered globular structures apparent on zoospore bodies under both growth conditions (Fig. S4).

10.1128/mBio.00832-21.5 FIG S4 Possible cellulosomes observed on the surface of *N. californiae* zoospores. (A to C) When grown on glucose, *N. californiae* zoospores imaged by HIM appear to show a relatively smooth surface with clusters of globular structures, indicated by the white arrow in panel C. In contrast, a zoospore imaged by HIM from a culture grown on corn stover has a much rougher surface, characteristic of the presence of many more surface proteins, which may be cellulosomes (D and E). It remains possible that this change in surface roughness is characteristic of different stages of zoospore development, but the increased deployment of surface-displayed cellulosomes would be consistent with the upregulation of degradative machinery observed for anaerobic fungi cultured on substrates more complex than glucose (32). The presence of surface-displayed globular structures under both glucose and corn stover growth conditions is also consistent with the immunofluorescence results presented in Fig. 5, suggesting that these structures may indeed be cellulosomes. Download FIG S4, DOCX file, 1.2 MB. Copyright © 2021 Lillington et al. 2021 Lillington et al. https://creativecommons.org/licenses/by/4.0/ This content is distributed under the terms of the Creative Commons Attribution 4.0 International license.

## DISCUSSION

Immunofluorescence microscopy with antibodies generated against the fungal dockerin domain and GH48 domain provided a useful tool to visualize cellulosome and CAZyme localization in anaerobic fungi grown on an array of carbon substrates. Our observation that cellulosome-associated dockerin domains and GH48 catalytic domains are highly localized to the rhizoid networks of mature *Piromyces finnis* cells during growth on a cellulosic substrate is strong evidence this rhizoidal cell morphology is an important driver of plant matter deconstruction by anaerobic fungi in the rumen. Additionally, our localization data for both the GH48 and dockerin domains is consistent with a prior study of other anaerobic fungal species, which found that unlysed cell pellets possessed cellulolytic activity comparable to that of the culture supernatant, suggesting surface display of cellulolytic enzymes (34). Given these results and the known genotypic and phenotypic similarities among anaerobic fungi, we speculate that cellulosome proteins localize to the rhizoids of rhizoid-forming anaerobic fungi from other genera besides *Piromyces* as well. While HIM micrographs captured cellulosome-like structures on the rhizoid networks of mature fungal cells, we were unable to address how fungal cellulosomes are attached to cell structures with our failure to observe colocalized ScaA and dockerin or GH48 signal in fixed cell samples. An alternative strategy to address this gap is likely needed, since the anti-ScaA antibody target shares homology with many classified scaffoldins, a large group of proteins encompassing a wide size range that also includes polypeptides with and without transmembrane anchors.

In addition to mature fungal rhizoids, zoospore surfaces also showed intense dockerin and GH48 localization. Some of the earliest investigations of anaerobic fungi following their initial discovery found that cell lysates from captured zoospores of three different genera all contained many of the enzymatic activities present in the fungal supernatant after growth, including cellulase and hemicellulase activity, highlighting the potential importance of this life stage in the biomass degradation process (35). Our data, consistent with this previous evidence that motile zoospores play a degradative role, provide the first evidence that zoospores also display cellulosomes on their surfaces, suggesting a broader physiological role for cellulosomes, which likely assist with both substrate attachment and degradation, as is the case with cellulolytic bacteria (36–38).

A particularly interesting finding of our work is that fungal zoospores, in contrast to thalli and zoosporangia, display cellulosomes during growth on simple soluble substrates such as glucose and cellobiose. Previous work demonstrated that culture supernatants from glucose- or cellobiose-grown cultures contained significantly less CAZyme activity and abundance than those from cells grown on lignocellulosic substrates (32). It was subsequently shown by bulk RNA sequencing that CAZyme expression in three strains of anaerobic fungi is strictly catabolite repressed by these simple carbohydrates (39). However, RNA from cells at all stages of the anaerobic fungal life cycle was used in these analyses, confounding any differences in gene expression response by cells at different life stages. This immunofluorescence microscopy approach provides the first evidence that production and display of major cellulosome protein domains is regulated differently by zoospores and thalli or sporangia. While we could not quantitatively measure changes or lack thereof in abundance of labeled zoospore surface proteins between growth conditions, the decrease in rhizoid-localized dockerin and GH48 to subdetectable levels during growth on glucose or cellobiose relative to lignocellulose supports the hypothesis that major cellulosome protein production may be uniquely constitutive in zoospores, regardless of growth conditions. This hypothesis would partially explain why a small subset of CAZyme- and dockerin-encoding genes showed no expression level change when sequencing the transcriptomes of *P. finnis* cells grown on glucose versus lignocellulose (32). Indeed, separate transcriptomic analysis of zoospores and sporangia of a parasitic chytrid, Batrachochytrium dendrobatidis, under the same environmental conditions found that more than half of the genes in the genome exhibited differential expression between the two life stages, including several peptidase gene families involved in pathogenicity, emphasizing the importance of cellular life stage in chytrid expression patterns and phenotype (40).

The potential importance of the anaerobic fungal life cycle in CAZyme production by these organisms is also reflected in the shifting cellulosomal CAZyme localization from zoospore body to mature cell rhizoid that was concomitant with cell maturation. As part of their development, monocentric anaerobic fungal zoospores shed or absorb their flagella, encyst, and then germinate to form rhizoids that grow into plant material, while the nucleus-containing zoospore body develops into the sporangium (2, 6). It is logical that fungal thalli direct their cellulolytic machinery to the rhizoids after attachment to a carbon source, but the cell biology of protein trafficking and secretion in anaerobic fungi has not been studied, though it is clearly of great importance given these organisms’ prolific enzyme production when cultured on lignocellulosic substrates (32). Recent work has demonstrated that rhizoids of chytrid fungi closely resemble hyphae of filamentous fungi (*Ascomycota*), which are better characterized (41, 42). Enzyme secretion in filamentous fungi such as Aspergillus niger is known to occur predominantly at the hyphal tips in a growth-coupled process (21); inhibition of processes that enable hyphae growth, such as actin polymerization, significantly reduced enzyme secretion and localization to the extracellular cell wall (43). Addition of the actin polymerization inhibitor cytochalasin B or a cell wall synthesis inhibitor, caspofungin, similarly stunted rhizoid growth in the chytrid Rhizoclosmatium globosum, but changes in protein secretion were not investigated (41). Given these established similarities, we hypothesize that CAZyme secretion is coupled to rhizoid growth during culturing on insoluble substrates, making the growing thallus life stage of interest for optimizing protein production by anaerobic fungi.

### Conclusions.

In summary, immunofluorescence microscopy of native anaerobic fungal cultures using antibodies raised against major fungal cellulosome protein domains provided strong evidence for the cell-associated *in vivo* localization of these molecular machines under different growth conditions. We confirmed the importance of rhizoids as centers for fungal cellulosome localization and biomass hydrolysis in mature fungal cells and established foundational data that zoospores display cellulosome proteins, paving the way for future study of how cellulosomes impact zoospore biology. Our approach also avoided the difficulties inherent in traditional omics-type analysis of organisms with complex life cycles to uncover uniquely constitutive production of cellulosome components by zoospores regardless of substrate complexity. These findings highlight how life cycle-dependent cell morphology and cellulosome localization contribute to biomass degradation by anaerobic fungi and, importantly, provide new support for the significance of zoospores in that process. Elucidating the uncharacterized regulatory relationships between life cycle progression and cellulosome production and function will benefit both laboratory efforts to engineer fungal cellulosomes for nanobiotechnology and industrial efforts to realize anaerobic fungi as platforms for bioprocessing. Higher-resolution structural biology studies detailing how cellulosomes orient enzyme active sites with different chemistries are still needed and will complement these efforts nicely. Such insights into the natural system will ultimately be key in the successful deployment of anaerobic fungi or their cellulosomes in industrial biotechnology.

## MATERIALS AND METHODS

### Cell culture and fixation.

Anaerobic fungal isolates were grown anaerobically under a headspace of 100% CO_2_ at 39°C in Hungate tubes containing CO_2_-flushed medium C (44) supplemented with various carbon sources at 1% (wt/vol) for insoluble substrates and 0.5% (wt/vol) for soluble substrates. Fungal cultures were passaged every 3 to 7 days to maintain viable cell populations by diluting 1 ml of growing culture into 9 ml of fresh medium containing a carbon source. Cultures harvested for microscopy were incubated for 3 to 4 days after inoculation. For all samples, fungal colonies were scraped off the Hungate tube walls, and the entire tube contents were transferred to a 15-ml Falcon tube. Fungal cells (and insoluble substrate if present) were pelleted in a fixed-angle rotor at 3,000 × *g* for 3 min and resuspended in cold phosphate-buffered saline (PBS) with 4% (wt/vol) formaldehyde. After fixation for at least an hour at 4°C, samples were washed once with an equal volume of PBS to remove excess formaldehyde and stored in PBS at 4°C prior to analysis.

### Antibody production.

Genes encoding each of the three key protein domains (ScaA fragment, dockerin, and GH48) were cloned into the pET-28a vector (Addgene) for expression of the target with N- and C-terminal 6× His tags in Escherichia coli BL21(DE3). For the ScaA fragment, the full gene product was used for animal immunization and antibody generation. Anti-dockerin and anti-GH48 antibodies were generated by animal immunization with synthetic 15-mer peptides taken from representative full dockerin and GH48 sequences. The three amino acid sequences used for antibody generation are included in Table S1. Purified protein product for immunization was prepared by protein expression and cell lysis followed by immobilized metal affinity chromatography (IMAC). Briefly, E. coli strains were grown at 37°C in Luria-Bertani (LB) medium supplemented with 50 μg/ml kanamycin. Protein synthesis was induced when the cells reached an absorbance at 600 nm of ∼0.6 by adding 0.1 mM isopropyl-β-d-thiogalactopyranoside (IPTG) to the medium. Cultures were incubated at 30°C overnight (16 to 24 h) following induction. To harvest protein product, cells were pelleted by centrifugation at >3,000 × *g* for >10 min and resuspended in 1× PBS plus 10 mM imidazole (pH 7.4) at 1% of the original culture volume. Cells were lysed by vortexing rigorously with 0.5-mm silica beads and soluble supernatant recovered by centrifugation at 10,000 × *g* for 10 min. 6× His-containing protein was purified from the soluble supernatant using HisPur nickel-nitrilotriacetic acid (Ni-NTA) resin following the manufacturer’s instructions (Thermo Fisher Scientific).

10.1128/mBio.00832-21.1 TABLE S1 Amino acid sequences of gene products used for antibody generation. Boldface sequences within the dockerin and GH48 represent the peptides used for animal immunization. The full-length protein was used for ScaA. Download Table S1, DOCX file, 0.01 MB. Copyright © 2021 Lillington et al. 2021 Lillington et al. https://creativecommons.org/licenses/by/4.0/ This content is distributed under the terms of the Creative Commons Attribution 4.0 International license.

Purified protein products were sent to GenScript as needed for antibody generation. All antibodies were verified with enzyme-linked immunosorbent assay (ELISA) titers of ≥64,000.

### Western blotting.

Cellulosome proteins isolated from *P. finnis* cultures as described in reference 10 were separated by SDS-PAGE and subsequently blotted onto a polyvinylidene difluoride (PVDF) membrane using a Bio-Rad TransBlot Turbo transfer system (Bio-Rad Laboratories, Hercules, CA). The membrane was then blocked with Tris-buffered saline plus 0.1% Tween 20 (TBS-T) supplemented with 5% milk powder for 1 h at room temperature. After being washed with TBS-T three times, the membrane was incubated with primary antibody for 1 h at 4°C. To perform the antigen blocking experiment, the membrane, containing identical protein samples, was split in two. Primary antibody (1 μg/ml) in TBS-T was used to label the control half, while 1 μg/ml primary antibody preincubated with 5 μg/ml immunizing peptide (sequence in Table S1) for 1 h prior to labeling was used to label the other half. Both halves were subsequently labeled with goat anti-rabbit horseradish peroxidase (HRP)-conjugated secondary antibody (Thermo Fisher Scientific number 31460). Blots were then developed with enhanced chemiluminescence (ECL) blotting substrate (Thermo Fisher Scientific number 32209) and imaged using a ChemiDoc imaging system (Bio-Rad Laboratories, Hercules, CA).

### Immunofluorescence microscopy.

Formaldehyde-fixed samples were washed three times in PBS for 10 min, blocked with 1% bovine serum albumin (BSA) for 1 h, and incubated with primary antibody overnight at 4°C. Antibody specifications were as follows: anti-ScaA, mouse monoclonal, 10 μg/ml in PBS; anti-dockerin and anti-GH48, rabbit polyclonal each at 10 μg/ml in PBS plus 1% BSA. After primary antibody incubation, samples were washed with PBS and incubated at room temperature for 1 h with a corresponding secondary antibody—goat anti-mouse Alexa Fluor 647 (Thermo Fisher), donkey anti-rabbit IgG Alexa Fluor 488 (Thermo Fisher), or donkey anti-rabbit IgG Alexa Fluor 594 (Thermo Fisher) at a 1 μg/ml concentration in PBS. Unless otherwise specified, samples were labeled with both anti-GH48 and anti-dockerin antibodies, necessitating a multistep labeling process. After the first labeling step, dually labeled samples were washed 3 times in PBS followed by fixation with 4% paraformaldehyde (Electron Microscopy Sciences, Inc.) for 10 min at room temperature. The above-described labeling process was repeated with appropriate primary and secondary antibodies. After immunolabeling, the samples were washed 3 times with PBS and finally counterstained with calcofluor white stain (Sigma-Aldrich). Confocal microscope images were acquired at 1-μm z-steps on a Zeiss LSM 710 scanning head confocal microscope with a Zeiss Plan Apo 40×/1.1 objective. Excitation lasers were 405, 488, 561, and 633 nm for the blue, green, orange, and red emission channels, respectively. Laser dwell times were 0.79 μs for each channel. Image processing was completed with Zen (Zeiss), ImageJ (NIH), and Volocity (Quorum Technologies Inc.).

### Cryosectioning.

Paraformaldehyde-fixed samples were placed into cryomolds, embedded in Tissue-Plus optimum cutting temperature (OCT) compound (Thermo Fisher), and then frozen at −20°C. Thin sections were generated on a CryoStar NX70 cryostat (Thermo Fisher) and placed onto a number 1 coverslip. The thin sections were then stained with 5 μg/ml FM 1-43 membrane stain (Thermo Fisher) and 1× SYBR gold nucleic acid stain (Thermo Fisher). Tiled confocal microscope images were acquired at 2-μm z-steps on a Zeiss LSM 710 scanning head confocal microscope with a Zeiss Plan Apo 40×/1.1 objective. Excitation lasers were 405 and 488 nm for the blue and green emission channels, respectively. Laser dwell times were 2.55 μs for each channel. Image processing was completed with Zen software (Zeiss).

### Helium ion microscopy.

Helium ion microscopy experiments were performed as described in reference 45. Specifically, fungi grown on various substrates were chemically fixed with 2% glutaraldehyde (Sigma-Aldrich), dehydrated through a series of 10-ml step gradients from 0% to 70% ethanol, and then centrifuged at 4°C (3,000 × *g* for 2 min). Samples were washed twice more with 10 ml of 100% ethanol for 15 min, centrifuged, and finally resuspended in 5 ml of 100% ethanol to remove any residual water. Fungal and/or plant biomass suspensions in 100% ethanol were gently extracted by wide-mouth pipet and placed onto stainless steel carriers for automatic critical-point drying (CPD) using an Autosamdri-815 (Tousimis, Rockville, MD), with CO_2_ as a transitional fluid. The CPD-processed biomass was mounted on aluminum stubs and sputter coated with approximately 10 to 20 nm of conductive carbon to preserve the sample surface information and minimize charge effects. Secondary electron images of the samples were obtained using an Orion helium ion microscope (HIM) (Carl Zeiss Microscopy, Peabody, MA) at 25 or 30 keV beam energy, with a probe current range of 0.1 to 1 pA. Prepared samples were transferred into the HIM via a load-lock system and were maintained at ∼3 × 10^−7^ torr during imaging. Use of a low-energy electron flood gun (∼500 eV) was applied briefly, interlaced with the helium ion beam, which enabled charge control to be maintained from sample to sample. The image signal was acquired in line-averaging mode, with 16 lines integrated into each line in the final image with a dwell time of 1 μs at a working distance range of 7 to 8 mm. Charge neutralization was applied to the sample after each individual line pass of the helium ion beam, which displaced charges on the surface and minimized charging effects in the final image. No postprocessing procedures were applied to the digital images besides standard noise reduction, brightness, and contrast adjustment using Photoshop plugins.
