# Supplementary material for: Cellulosome Localization Patterns Vary across Life Stages of Anaerobic Fungi
Source: mBio. 2021 Jun 1;12(3):e00832-21. doi: 10.1128/mBio.00832-21 (PMC8262932; doi:10.1128/mBio.00832-21)

**Figure S1. Potential cellulosome structures from *Piromyces finnis* visualized by Helium Ion Microscopy (HeIM).** Images were obtained from fixed, dehydrated samples of *P. finnis* grown on reed canary grass as described in the Materials and Methods. Globular structures with diameters in the 10 nm to 100 nm range, consistent with MDa-sized protein complexes, are apparent on the surface of filamentous structures ~10 nm in diameter. Filaments this small could possibly originate from the reed canary grass substrate, in which case the globular structures may be bound, cell-free cellulosome complexes.


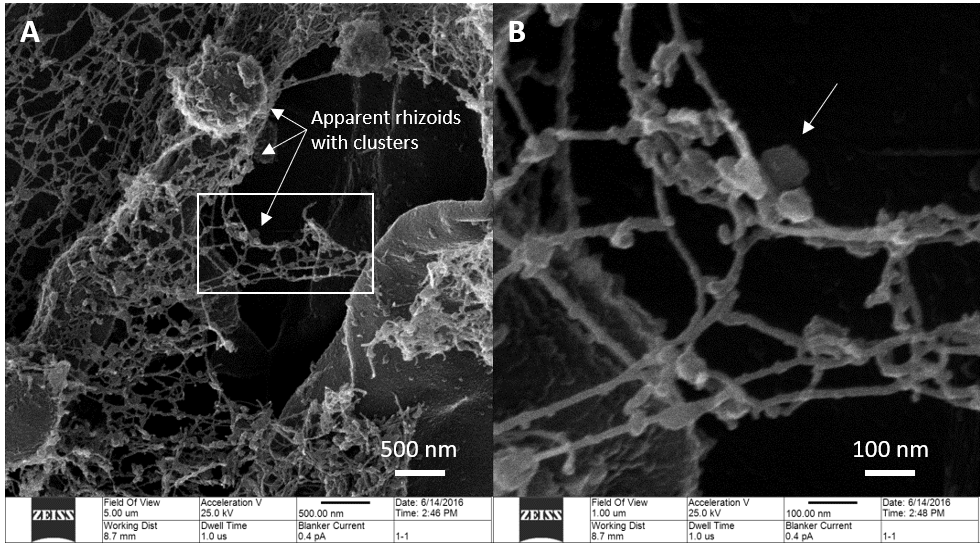

Supplement: FIG S1 [file mbio.00832-21-sf001.docx]
