# Supplementary material for: Cellulosome Localization Patterns Vary across Life Stages of Anaerobic Fungi
Source: mBio. 2021 Jun 1;12(3):e00832-21. doi: 10.1128/mBio.00832-21 (PMC8262932; doi:10.1128/mBio.00832-21)

**Figure S2. Larger rhizoids of *Piromyces finnis* and *Neocallimastix californiae* cells have rough surfaces covered with globular structures that may be proteins or protein complexes.** Panels A-C show micrographs from *P. finnis* cultured on reed canary grass, in which B is a magnified close-up of part of the rhizoid annotated in A).


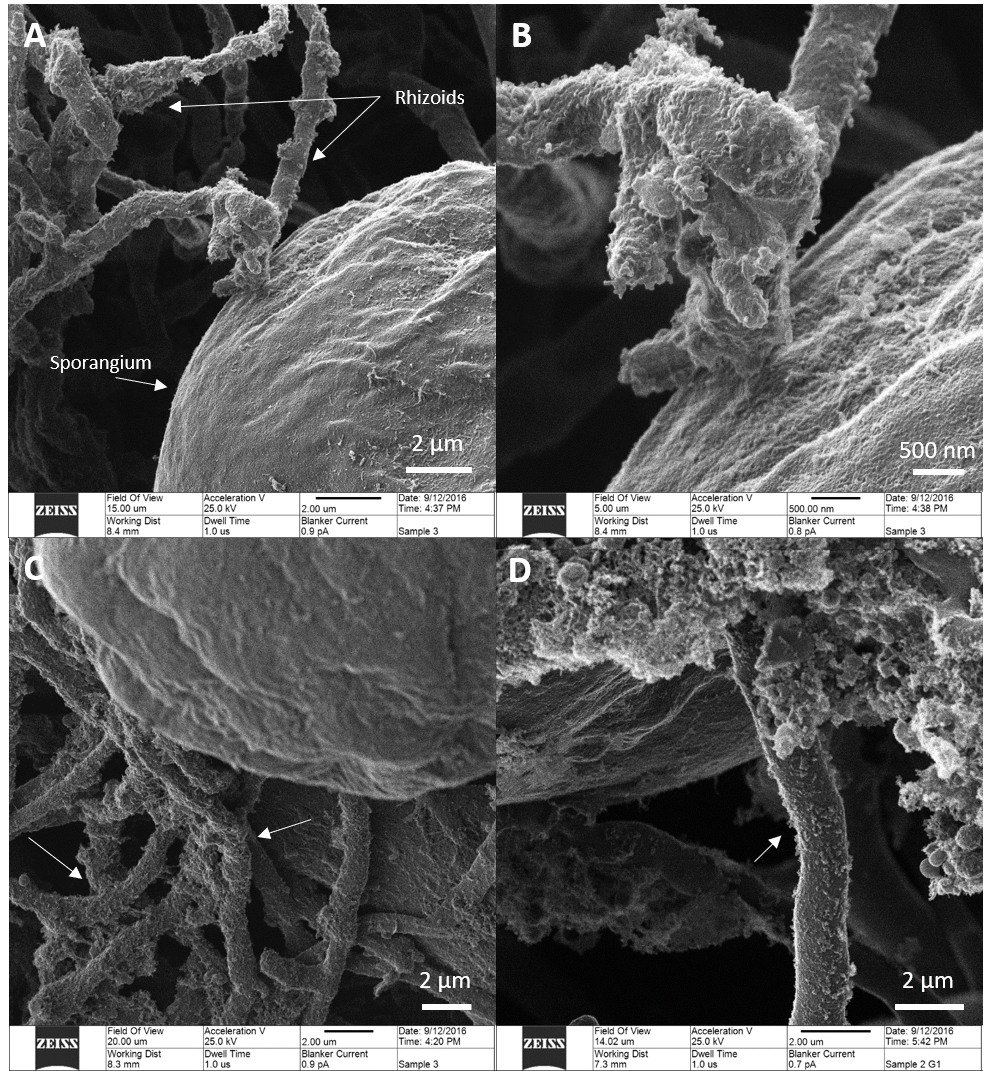

Supplement: FIG S2 [file mbio.00832-21-sf002.docx]
