# Supplementary material for: Cellulosome Localization Patterns Vary across Life Stages of Anaerobic Fungi
Source: mBio. 2021 Jun 1;12(3):e00832-21. doi: 10.1128/mBio.00832-21 (PMC8262932; doi:10.1128/mBio.00832-21)

**Figure S3. Reed canary grass from culture tubes not inoculated with anaerobic fungi show whispy, complex microstructure by Helium Ion Microscopy.** A) Fibrillar microstructures with diameters around 10 nm are present in biomass-only control samples, making it more difficult to identify potential parts of fungal cells or cellulosomes at this length scale. B-D) Larger, cylindrical structures that resemble anaerobic fungal rhizoids also appear in the biomass-only control, emphasizing both the potential uncertainty in identifying sub-cellular structures from HeIM images and the need to identify rhizoids as structures emerging from sporangia, which are clearly observed only in the fungi-containing samples.


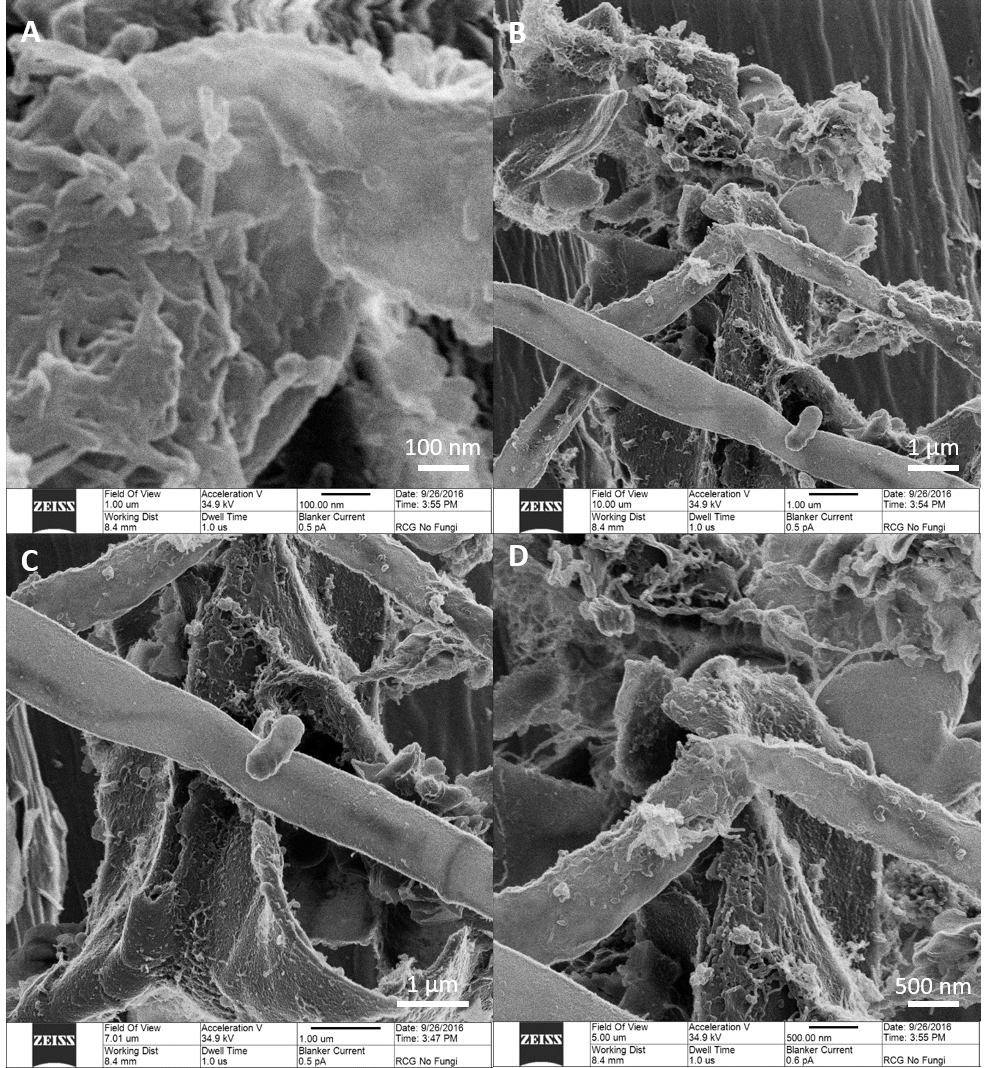

Supplement: FIG S3 [file mbio.00832-21-sf003.docx]
