# Supplementary material for: Cellulosome Localization Patterns Vary across Life Stages of Anaerobic Fungi
Source: mBio. 2021 Jun 1;12(3):e00832-21. doi: 10.1128/mBio.00832-21 (PMC8262932; doi:10.1128/mBio.00832-21)

**Figure S4. Possible cellulosomes observed on the surface of *N. californiae* zoospores. (**A-C) When grown on glucose, *N. californiae* zoospores imaged by HeIM appear to show a relatively smooth surface with clusters of globular structures as annotated by the white arrow in panel C). In contrast, a zoospore imaged by HeIM from a culture grown on corn stover has a much rougher surface, characteristic of the presence of many more surface proteins, which may be cellulosomes (D-E). It remains possible that this change in surface roughness is characteristic of different stages of zoospore development, but the increased deployment of surface-displayed cellulosomes would be consistent with the upregulation of degradative machinery observed for anaerobic fungi cultured on substrates more complex than glucose (K. Solomon et al, *Science*, **2016**). The presence of surface-displayed globular structures under both glucose and corn stover growth conditions is also consistent with the immunofluorescence results presented in Figure 5, suggesting these structures may indeed be cellulosomes.


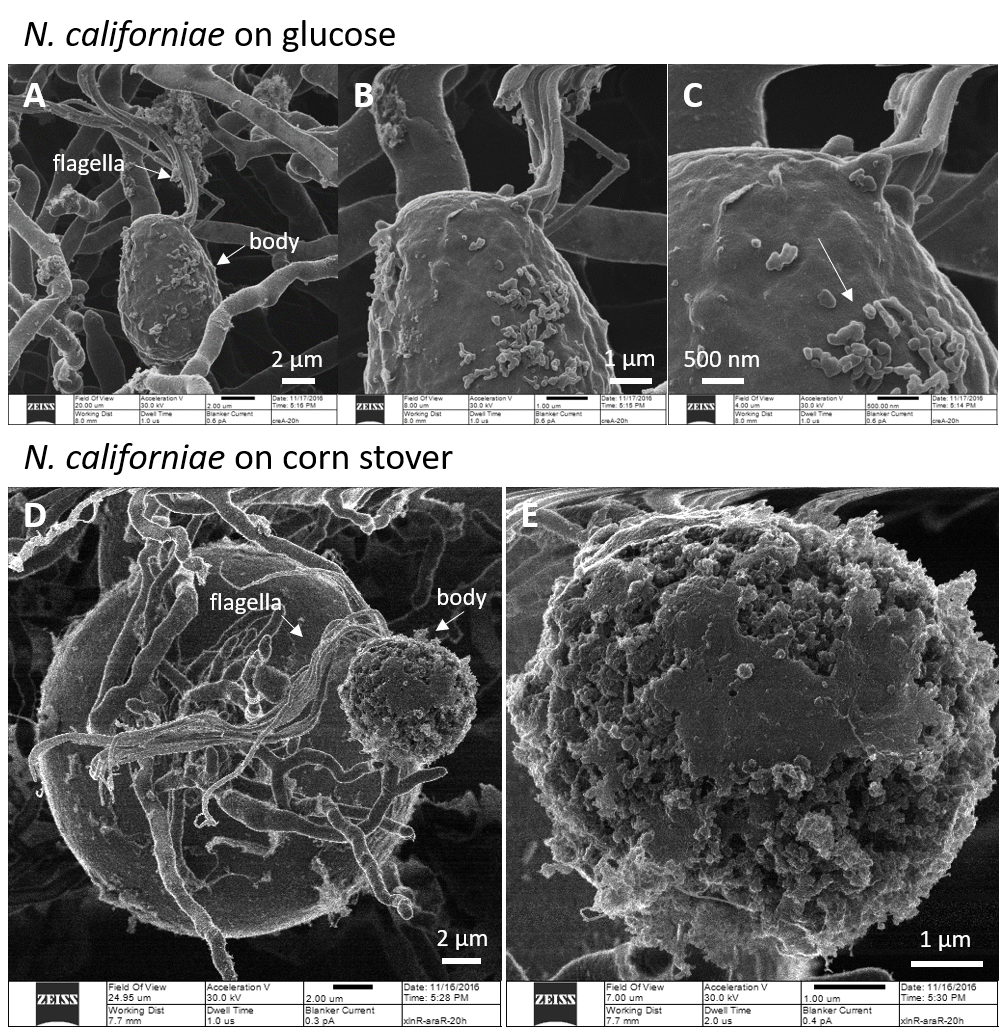

Supplement: FIG S4 [file mbio.00832-21-sf004.docx]
