# Supplementary material for: Cellulosome Localization Patterns Vary across Life Stages of Anaerobic Fungi
Source: mBio. 2021 Jun 1;12(3):e00832-21. doi: 10.1128/mBio.00832-21 (PMC8262932; doi:10.1128/mBio.00832-21)
Supplement: TABLE S1 [file mbio.00832-21-st001.docx]

**Supplementary Information: Cellulosome localization patterns vary across life stages of anaerobic fungi**

Stephen P. Lillington^1^, William Chrisler^2^, Charles Haitjema^1^, Sean Gilmore^1^, Chuck R. Smallwood^2^, Vaithiyalingam Shutthanandan^2^, James E. Evans^2^, and Michelle A. O’Malley^1*^

^1^Department of Chemical Engineering, University of California, Santa Barbara, Santa Barbara, CA, 93106, United States

^2^Environmental Molecular Sciences Laboratory, Pacific Northwest National Laboratory, Richland, WA 99352, United States

*Corresponding author. Email: [momalley@ucsb.edu](mailto:momalley@ucsb.edu)

**Table S1.** Amino acid sequences of gene products used for antibody generation. Bolded sequences within the dockerin and GH48 represent the peptides used for animal immunization. The full length protein was used for ScaA.

| **Protein of interest** | **Sequence** |
| --- | --- |
| *P. finnis* ScaA fragment | NENNKIGYYLGYYNSYLIKCSDNGNCEKEILDDSAI  GYYLNMDNSKPLIQCYTSLGKTNCAAVSEVKNGYYT  TPESTTDGNIIKCNNGKCKLETVTVTAQEIKYFVSG  ETNKHITKCEEVVEQEPEPLRRSGVEMVTKCTAIAS  PKLGWYVNGDKNAKTEGLDLILCSKTNGSFSCIEKP  KNIGYYLSSEDNKLLNCDKTGCNDVVDLSESSAN |
| *P. finnis* dockerin | MKCWATSLGYPCCKEA**NPIIFYKDEDGDWGC**ENNS  WCGIIKNEKPACSEKIINQGYPCCSKETQVYYTDE  SGEWGVENNNWCGILN |
| *P. finnis* GH48 | MPSIRSALALLGATAAIAAP**MQKRANDEYGRRVTC**LY  DVMTGNGSYSSEYFSAEKVPYHSVETLMVEAPDHGHE  SVSETFSFWIWLEAVNGKITGNYDGVEEAWNYLEKHI  IPDSKNQPGNSKYNPSSPATYAAEHDDIDGYPSKLIF  QDGIVGEDPIAKELSQAYGNWDIYIMHWIIDGDNWYG  YGQQGDGTSKPSFINTFQRGASESTWKTVPHPCWEAM  KWGGRNGFLDLFTVDNSYAKQWRYTAAPDADARAVQA  AYFAYIWAEEDGVNLSSVAAKAAKLGDYLRYAQYDKY  FKKIGNCVGSDKCSAGRGKNSAHYLISWYFAWGGGLQ  GDWAWRIGSSHTHSGYQNPLAAWILSTQAAFKPKSST  GAKDWATSMDRQLELFRWLQTPEGCIAGGATNSWQGS  YAQPSSDITTFYGMWYDWQPVYHDPPSNNWTGMQGWG  MERVCSLYYVSGDERAGKVCQEWAKWVKDTTRVSGGE  IVHATNLDWSGNPDEWNSSNFNKSNLNRSLHGTVSSE  GVDLGSIASIIKGLMWVSMRDGDQEGINLSIEVMDAI  ENYKDDLGYSSHEARGDYDKFGGEVYIPSGWTGKNAQ  GANIKSGVTFIDIRPKYKQDPDWAQVEEYLNGGNPPE  FNYHRFWAQTEIAVANGLISIYGLQKDGSSNNDNNNS  SNETTV |
